# Patient-reported outcome measure (PROM) programs for monitoring symptoms among patients treated with immunotherapy: a scoping review

**DOI:** 10.1093/jncics/pkae102

**Published:** 2024-10-28

**Authors:** Sylvie D Lambert, Sara Soldera, Jordana Kazdan, Francesca Frati, Anita Slominska, Melina Boutin, Vanessa Samouelian, Caroline Letendre, Karine Bilodeau, Doris Howell, Karine Le Breton, Michel-Olivier Gratton

**Affiliations:** St Mary’s Hospital Research Centre, Montreal, QC H3T 1M5, Canada; Ingram School of Nursing, McGill University, Montreal, QC H3A 2M7, Canada; Medical Oncology, Cedars Cancer Centre, McGill University Health Centre, Montreal, QC H4A 3J1, Canada; St Mary’s Hospital Research Centre, Montreal, QC H3T 1M5, Canada; Schulich Library of Physical Sciences, Life Sciences, and Engineering, McGill University, Montreal, QC H3A 0C1, Canada; St Mary’s Hospital Research Centre, Montreal, QC H3T 1M5, Canada; Centre Intégré de Cancérologie de la Montérégie, Hôpital Charles-Lemoyne, Greenfield Park, QC J4V 2G9, Canada; Faculté de Médecine et des Sciences de la Santé, Université de Sherbrooke, Sherbrooke, QC J1K 2R1, Canada; Centre intégré de cancérologie, Le Centre Hospitalier de l’Universite de Montreal, Montréal, QC H2X 0C1, Canada; Department of Hematology and Oncology, Integrated university health and social services centres (CIUSSS) de l’Est-de-L’Île-de-Montréal—Hopital Maisonneuve-Rosemont, Montreal, QC H1T 2M4, Canada; Faculty of Nursing, University of Montreal, Marguerite-d’Youville Pavilion, Montreal, QC H3T 1A8, Canada; Centre de Recherche, Hôpital Maisonneuve-Rosemont Research Centre, Montreal, QC H1T 2M4, Canada; University Health Network, Toronto, ON M5G 2C4, Canada; Faculty of Nursing, University of Montreal, Marguerite-d’Youville Pavilion, Montreal, QC H3T 1A8, Canada; Department of Hematology and Oncology, Integrated university health and social services centres (CIUSSS) de l’Est-de-L’Île-de-Montréal—Hopital Maisonneuve-Rosemont, Montreal, QC H1T 2M4, Canada

## Abstract

**Background:**

Monitoring toxicities among patients receiving immune checkpoint inhibitors using patient-reported outcome measures (PROMs) is relatively recent. This scoping review aims to guide decision making in the development of PROMs programs for patients receiving immune checkpoint inhibitor therapy.

**Methods:**

Four electronic databases were searched from inception to January 2024. Data on PROM programs for patients receiving immune checkpoint inhibitors (eg, PROMs used, frequency) were extracted. Two authors with established interrater reliability screened titles, abstracts, and full texts. A narrative synthesis identified patterns in the data.

**Results:**

A total of 22 articles described 16 unique multicomponent, electronic PROM programs for patients receiving immune checkpoint inhibitor therapy, mainly developed for remote monitoring of toxicities between appointments. Patients typically completed 18-26 items from the Patient-Reported Outcomes Common Terminology Criteria for Adverse Events (PRO-CTCAE) or Common Terminology Criteria for Adverse Events (CTCAE) weekly, with high adherence and satisfaction. Commonly monitored symptoms were diarrhea, fatigue, shortness of breath, cough, nausea, decreased appetite, rash, joint pain, pain, and mood. Other features of PROMs programs included clinician alerts, with some programs only flagging symptoms that had an impact on treatment. Some programs also or only sent alerts to patients to contact their clinicians and gave access to symptom management information. In terms of efficacy, the only consistent finding was an increase in quality of life.

**Conclusions:**

The findings of this scoping review provide some indication as to which components of a PROM program are promising. However, as the evidence base for using PROMs among patients receiving immune checkpoint inhibitors is growing, many questions remain, including which symptoms to monitor, using which PROM, and at what frequency. More trials are needed to answer these questions and to determine how best to implement PROMs among patients receiving immune checkpoint inhibitor in clinical practice.

## Introduction

Immune checkpoint inhibitors are becoming standard of care for various cancers.^1^ The use of immune checkpoint inhibitors has improved survival outcomes in the curative and advanced settings for patients with melanoma, head and neck cancer, Hodgkin lymphoma, non–small cell lung cancer, and many others.^2-7^ These humanized monoclonal antibodies target the immune checkpoint proteins, such as programmed cell death protein 1 (PD-1) and cytotoxic T-lymphocyte associated protein 4 (CTLA-4), that act as co-inhibitory receptors negatively regulating T-cell mediated immune responses. By inhibiting the interaction between these proteins and their respective ligands, immune checkpoint inhibitors reactivate the immune response directed at tumor cells that previously exploited this mechanism of immune tolerance.^1^

Despite immune checkpoint inhibitors’ encouraging survival outcomes, harnessing the immune system to treat tumor cells is, however, inherently associated with a risk of a unique set of toxicities referred to as immune-related adverse events. Frequent immune-related adverse events include fatigue, diarrhea, rash, pruritus, and endocrinopathies (eg, hypo- and hyperthyroidism).^8^^,^^9^ Rarer toxicities that can be potentially life threatening include hepatotoxicity, pneumonitis, myocarditis, nephritis, and various neurotoxicities.^8^^,^^9^ The incidence of any grade immune-related adverse event, as defined by the Common Terminology Criteria for Adverse Events (CTCAE), is between 17% and 53%.^10^ Grade 3 or more immune-related adverse events occur within the range of 5%-42%, being more common for anti–CTLA-4 drugs,^11^ and can reach 55% with combination CTLA-4 and PD-1 therapies.^12^ Lower-grade immune-related adverse events are often reversible and usually do not require treatment discontinuation. However, moderate to severe immune-related adverse events can cause irreversible organ damage, adversely impacting patients’ quality of life (QOL) and survival.^11^

The unpredictable nature, duration, and severity of immune-related adverse events require close symptom monitoring,^13^ as minor changes in symptoms sometimes represent early signs of potentially severe immune-related adverse events.^8^ Unlike chemotherapy, immune-related adverse events are not dose dependent and can occur at any time during treatment, even when the immune checkpoint inhibitor was previously well tolerated and even after discontinuation.^14^ Immune checkpoint inhibitors are commonly combined with chemotherapy and targeted therapies,^15^ which have similar side effects that are managed very differently, further complicating the evaluation of immune-related adverse events.

The systematic collection of patient-reported outcomes is now recognized as an effective intervention for early symptom identification and management.^16^ Patient-reported outcomes are any report on patient’s health that comes directly from the patient,^17^ and are typically collected using short, validated measures (called patient-reported outcome measures or PROMs).^18^ PROMs data shed light on symptom severity from the patient’s perspective and alert clinicians to unrecognized and/or undertreated toxicities.^16^ This approach has been shown to reduce symptom burden, enhance patients’ QOL, improve survival, and reduce health-care service use.^19^ PROMs have been shown to be more predictive of overall survival compared with established measures including the Eastern Cooperative Oncology Group Performance Status and the Lung Immune Prognostic Index.^20^ However, whereas PROMs have been implemented mainly for patients receiving radiotherapy and/or chemotherapy, their adaptation for patients receiving immune checkpoint inhibitors remains unclear.

A recent systematic review examined 5 electronic PROM programs for patients receiving immune checkpoint inhibitors, assessing their feasibility, acceptability, and impact.^21^ These screened for most immune-related adverse events per the American Society for Clinical Oncology guidelines.^22^ Although feasibility and acceptability were supported, their efficacy was mixed. The present review provides a complementary and broader summary of the evidence to guide decision making when developing PROM programs for patients receiving immune checkpoint inhibitors. Inspired by the International Society for Quality of Life Research’s User’s Guide for designing PROM programs,^23^ this scoping review reports on (1) types of PROM programs and their goals; (2) screened patient-reported outcomes and corresponding PROMs; (3) PROM administration (including frequency); (4) clinical workflow integration and implementation; (5) clinical responses to PROM data; and (6) evaluation of added value.

## Methods

This scoping review adhered to the Joanna Briggs Institute (JBI),^24^ Preferred Reporting Items for Systematic Reviews and Meta-Analyses (PRISMA)–Scoping Reviews, and PRISMA Statement guidelines^25^^,^^26^ and was registered on Open Science Framework (OSF) registries (10.17605/OSF.IO/AJ8TE).

### Inclusion and exclusion criteria

Eligible studies were qualitative or quantitative studies published (or in press) from 1986 to January 2024, involving adults with cancer, regardless of diagnosis or stage, receiving immune checkpoint inhibitors. Protocols were included for a comprehensive overview of upcoming studies. However, if a results paper was published, the protocol was excluded. Mixed treatment studies required at least 50% of patients on immune checkpoint inhibitors. Unspecified immunotherapy was included, if this did not include chimeric antigen receptor (CAR) T-cell therapies. Eligible studies reported on PROM programs, defined as any intervention leveraging PROMs to capture patients’ perspective on their health and providing real-time feedback to inform clinicians about patients’ health. Reviews, letters to the editor, and conference abstracts were excluded. Articles written in English or French were included. Prevalence studies of immune-related adverse events, correlation studies on patient-reported outcomes and health outcomes, and psychometric studies of PROMs were excluded. Immunotherapy among pediatric populations were also excluded as were studies on clinician grading of immune-related adverse events.

### Search strategy

Four bibliographic databases were searched using a 3-step iterative process (Ovid- Medline ALL 1946 onward, Ovid- Embase Classic plus Embase 1947 onward, Ovid- APA PsycINFO 1967 onward, and CINAHL Plus with full text). See the published search strategies here: https://borealisdata.ca/dataset.xhtml?persistentId=doi:10.5683/SP3/DBLSMA.

Step 1 involved a medical librarian (FF) and 2 authors (SL, SS) developing the initial Embase search strategy using a single-line method.^27^ The search combined subject headings and keywords pertaining to patient-reported outcomes and PROMs, cancer, and immune checkpoint inhibitors. No limits, restrictions, or filters were used. A second librarian peer-reviewed the search strategy using the PRESS (Peer Review of Electronic Search Strategies) guideline.^28^ Feedback was integrated, and the updated search was then translated into Medline and ran first on March 10, 2021. Duplicates were removed using EndNote^29^ and during title screening using the Rayyan web application.^30^

Step 2 involved the librarian revising the search based on bibliographic records obtained in step 1, adding additional relevant subject headings and keywords as needed.

Step 3 included translating the revised search into PsycINFO and CINAHL on December 17, 2021. Duplicates were removed using the Bond University’s Systematic Review Accelerator deduplicator tool^31^ and during screening using Rayyan.^30^ Searches were rerun on May 19, 2022, and January 16, 2024.

Secondary searches included ProQuest Dissertations and Theses Global on December 17, 2021; conference proceedings in Scopus on April 19, 2022; ClinicalTrials.Gov on April 12, 2022; and the reference list of all included full texts. Google Scholar, Scopus, and Web of Science Core Collection were also used to find studies citing the full texts. Authors of included studies were contacted for any new papers published in January 2024.

#### Interrater reliability

For the initial 2 databases, authors (SL, JK, MB, SS) screened 100 titles together, evaluating each title against the inclusion and exclusion criteria. Titles were considered if they mentioned symptom identification or screening, regardless of explicit mention of PROMs. If there was any doubt, the title was included, and its inclusion was further considered at the abstract stage. Then, authors independently screened another set of 100 titles to establish interrater reliability, aiming for at least 0.8 on the Gwet AC and Kappa coefficient.^32^ After each round of independent title screening, authors met to discuss discrepancies. Initial Kappa coefficient or Gwet AC was in the 0.7 range, and discussions led to refining inclusion criteria to focus on PROM programs. Two independent screening rounds were needed to reach 0.8. Interrater reliability was verified at the abstract stage (titles and abstracts screened separately) as well, the Kappa coefficient and the Gwet AC exceeded 0.8 on the first round. For the remaining databases, the interrater reliability was confirmed during title screening only. All included full texts were confirmed by 2 authors, and discrepancies were discussed at regular team meetings to reach consensus.

### Data extraction

Data were extracted using an Excel form based on the *Cochrane Handbook for Systematic Reviews of Interventions*.^33^ Similar data extraction forms have been used across other reviews by the team.^34-38^ Data extraction was led by 3 trained research assistants. Training included extracting 2 full texts together and then extracting independently. If some of the data were unclear, original authors were contacted. Data extraction was confirmed by the first author. Discrepancies were discussed and resolved at team meetings.

The data extraction form documented (if applicable) the following: (1) reviewers’ names; (2) authors and year; (3) country; (4) platform and software used; (5) cancer type; (6) immune checkpoint inhibitor treatment; (7) setting; (8) baseline sample size and attrition; (9) population; (10) confirmation of eligibility; (11) objectives; (12) theoretical framework; (13) design; (14) type of control group; (15) delivery of the PROM program (eg, provider, duration); (16) types of patient-reported outcomes and PROMs; (17) timing and frequency of PROMs; (18) format; (19) real-world implementation or trial; (20) who responded to the PROM data and how (eg, alert system used); (21) clinical and/or patient report; (22) key findings; (23) facilitators and barriers of patient-reported outcome screening; (24) limitations of study and clinical implications; and (25) future studies.

### Data analysis

This scoping review used a narrative synthesis approach. Columns in the Excel sheet pertaining to each of the objectives were analyzed in turn using content analysis to extract patterns and themes.^39^ Frequency count was also used, particularly to identify the components of the PROM programs.

## Results

**Figure 1.**
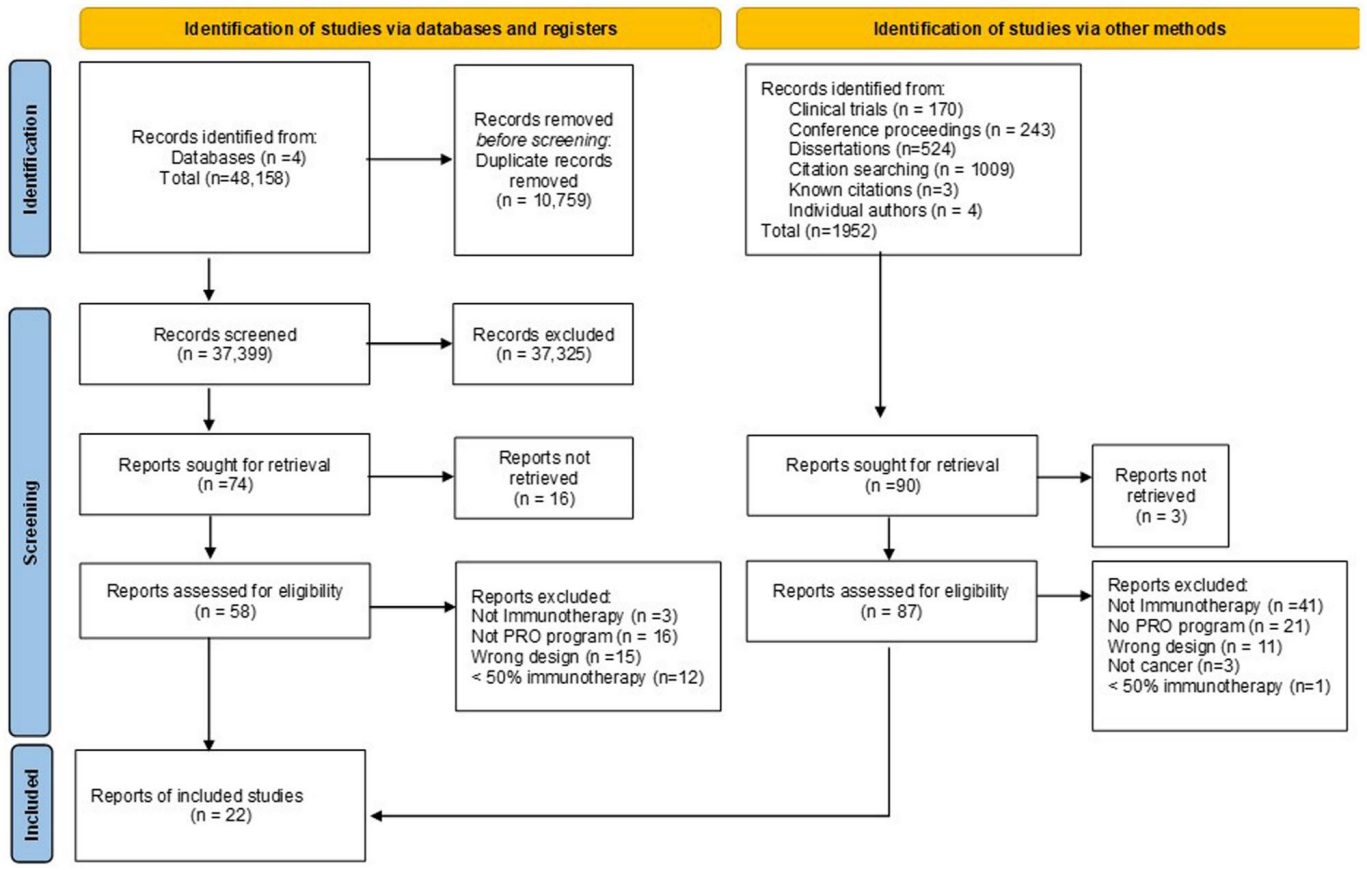
Preferred Reporting Items for Systematic Reviews and Meta-Analysis flowchart. PRO = patient-reported outcome.

According to the PRISMA flowchart (Figure 1), 145 full texts underwent eligibility assessment. Of these, 123 were excluded primarily because patients were not treated with immunotherapy or lacked a PROM program. The 22 studies included^40-61^ are summarized in Table 1 and encompass 16 unique PROM programs because of overlapping studies. Most studies took place in European countries.^40-45^^,^^47-53^^,^^56-58^^,^^60^^,^^61^ Study design included randomized controlled trials (RCTs),^50^^,^^51^^,^^55^ quasi-experimental trials (nonrandomized),^49^^,^^60^ feasibility studies,^41^^,^^46^^,^^56^ observational studies,^40^ mixed methods studies,^48^^,^^52^^,^^53^ protocols,^45^^,^^47^^,^^57^^,^^59^^,^^61^ machine-learning studies,^42-44^ and descriptions of the development of the PROM program.^54^^,^^58^

**Table 1.** Overview of studies (by design type)

Author, year country	Aim(s)	Methods	Overview of key components of PRO program	Key findings^a^
**Randomized Controlled Trials (RCTs)**				
Zhang, Zhang, Shen, et al.^55^ 2022 China	Compare the efficiency between PROMs and traditional follow-up models in improving the safety and QOL of patients receiving immunotherapy and reducing the time of follow-up sessions	Theoretical framework = not mentioned Setting = 28 tertiary care hospitals Population = mixed cancers Sample size (n = 278) Mean age = 57.6 y (T), 60.1 y (C) Female = 24.8% (T), 27% (C) Race and ethnicity = not reported Treatment = immune monotherapy 65.8%	PROMs = CTCAE-based plus images uploaded Mode = mobile application Duration = 6 months or until treatment completion Frequency = weekly Clinician summary report = not mentioned Patient summary report = not mentioned Alerts to clinicians = If grade 3 or 4 irAEs were reported, the model alerted HCPs Alerts to patients = not mentioned Patient education = grade 1-2 irAEs = automatic advice sent For serious irAEs = advised to go to emergency department	Incidence of grade 3-4 irAEs = T < C Emergency room visits = T < C QOL = 3 months T = C, 6 months T > C Mean time for follow-up = T < C Treatment discontinuation = T > C Death rate = T = C
Tolstrup, Pappot, Bastholt, et al.^51^ 2022 Denmark	Examine the impact of using electronic PROMs on patients’ QOL and associations between irAEs severity and QOL	Theoretical framework = not mentioned Setting = 1 cancer center Population = patients with metastatic melanoma Sample size (n = 146) Mean age = 66 y Female = 52% (T), 41% (C) Race and ethnicity = not reported Treatment type = mixed ICIs, most common pembrolizumab 52% (T), 49% (C)	PROMs = 29-item PROM (based on PRO-CTCAE) Mode = web platform Ambuflex, (tablet provided by the study) Duration = 24 weeks Frequency = weekly Clinician summary report = yes, but clinicians did not routinely monitor the patient report Patient summary report = no Alerts to clinicians = no, left to the patients to react to the alert Alerts to patients = only for symptoms that could become severe Patient education = none	QOL 24 weeks T = C, 48 weeks T > C Association between QOL and severe irAEs = none
Tolstrup, Bashtholt, Dieperink, et al.^50^ 2020 Denmark	Evaluate the impact of PROMs on the number of grade 3 or 4 irAEs during immunotherapy among patients with melanoma	Same as Tolstrup et al.^51^	Same as Tolstrup et al.^51^	78% of patients reported symptoms weekly Grade 3 or 4 irAEs = T = C Telephone consultations = T > C No. of extra hospital visits (including emergency room) = T = C No. of days in hospital = T = C No. of days on steroids = T > C
**Quasi-experimental trial**				
Skovlund, Vind Thaysen, Schmidt, et al.^49^ 2021 Denmark	Evaluate the potential of using PROMs as a dialogue-based tool in consultations	Theoretical framework = not mentioned Setting = Aarhus University Hospital (T), Odense University and Herlev Hospitals (C) Population = metastatic melanoma Sample size (n = 279) Median age = 65 y (T), 67 y (C) Female = 37% (T), 53% (C) Race and ethnicity = not reported Treatment = immunotherapy 73% (T), 70% (C)	PROMs = EORTC-QLQC30 plus Hospital Anxiety and Depression Scale (HADS) Mode = using web platform Ambuflex Duration = 12 months Frequency = before every consultation with a physician Clinician summary report = yes Patient summary report = no mention Alerts to clinicians = no mention Alerts to patients = no mention Patient education = no mention	Patient activation = T = C Quality of life = general T > C; melanoma specific T > C; physical T = C; social T > C; emotional T > C; functional T = C Self-efficacy = 12 months T = C Perceived efficacy in patient-physician interactions = T = C
Dickson, Beauchamp, Perry, et al.^60^ 2024 USA	Evaluate utilization of an electronic PROM platform	Theoretical framework = not mentioned Setting = not mentioned (historical control group used) Population = patients with solid tumor malignancy (mixed) Sample size = T=1014, C=538 Mean age = T=68.3, C=70.2 y Female = 39.9% (T), 38.1% (C) Race and ethnicity = White 88.5% (T), 90% (C) Treatment type = mixed ICIs, most common pembrolizumab	PROMs = PRO-CTCAE (No. of symptoms and type not reported) Mode = Noona Duration = 6 months Frequency = not mentioned Clinician summary report = not mentioned Patient summary report = not mentioned Alerts to clinicians = yes Alerts to patients = not mentioned Patient education = not mentioned	Adherence = 31% used the PRO platform Treatment discontinuation = for index treatment T < C, T = C for subsequent regimen Survival = 6 months T > C Usage = 56.9% PROMs answered, few symptoms reported (mean = 0.4-0.7), 6.2% reported 1-2 severe symptoms, 11.2% of patients with alerts, 0.2 (SD = 0.6) alerts per patients. The most frequent alert outcomes were resolved on phone per message and provider consulted
**Mixed methods**				
Tolstrup, Pappot, Bastholt, et al.^52^ 2020 Denmark	Examine the experiences of malignant melanoma patients and their treating clinicians with an electronic Health intervention based on PROMs	Theoretical framework = not mentioned Setting = Department of Oncology, Odense University Hospital Population = patients with metastatic melanoma Sample size = 57 of 70 who were randomly assigned to PROM program (patients), 5 (HCPs) Mean age = 65 y (patients), 43 y (HCPs) Female = 64.9% (patients), 100% (HCPs) Race and ethnicity = not reported Treatment type = pembrolizumab, nivolumab, or ipilimumab, or combination therapy	Satisfaction substudy of Tolstrup et al.^51^	Patient satisfaction questionnaire= none found intervention too time consuming; frequency of PROMs was just right; satisfaction high, but lowest satisfaction for use of patient responses in care; agreed that PROMs improve care Interviews with patients= high usability, minor technical challenges; half found PROMs reassuring; heightened attention to side effects, easier to remember symptoms; majority felt report was seen and used in consultation; made patients feel more involved Focus groups clinicians= clinicians did not find symptoms as severe as patients; use of PROMs in consultation found time consuming; agreed that patients were better prepared for consultations; still perceived as a valuable tool
Trojan, Huber, Brauchbar, et al.^53^ 2020 Switzerland	Explore real-world usability and acceptance of a smartphone app for PROMs among patients receiving ICI	Theoretical framework = not mentioned Setting = not mentioned Population = patients with advanced or metastatic PD-L1–positive cancer Sample size = 6 Median age = 62 y Female = 0% Race and ethnicity = not mentioned Treatment type = anti–PD-L1 checkpoint	Mode = Consilium app Duration = 12 weeks Frequency = daily Clinician summary report = yes Patient summary report = no Alerts to clinicians = no Alerts to patients = yes Patient education = yes	No. of symptoms = on average, 2.4 entries per day; patients reported between 4 and 16 symptoms No. of unplanned hospitalizations= 0 No. of telephone consultations= 6 No. of early blood controls= 3 No. of ambulatory clinical assessments= 3 Usability = high usability; good or very good effect on symptom management, doctor consultations, communication
Schmalz, Jacob, Ammann, et al.^48^ 2020 Germany	Assess patients’ and HCPs’ adoption of a Digital Patient Monitoring and Management tool	Theoretical framework = not mentioned Setting = 10 clinics in Germany, Switzerland, and Finland Population = patients with metastatic non–small cell lung cancer Sample size = 45 (13 nurses, 11 physicians, 27 patients) Mean age = 40-70 y Female = 40.7% (patients), 50% (physicians), 92.3% (nurses) Race and ethnicity = not mentioned Treatment type = immunotherapy	PROMs = 18 items from PRO-CTCAE Mode = Kaiku Duration ≥ 3 months Frequency = weekly Clinician summary report = yes Patient summary report = yes Alerts to clinicians = yes Alerts to patients = yes Patient education = yes	Patient adherence PROMs and alerts most used item Usability and satisfaction Patients and HCPs think the Digital Patient Monitoring and Management tool enabled more efficient and focused communication. Empowered patients to evaluate and monitor symptoms. Well integrated into clinical workflow. Saved time (reducing phone consultations) Workload most common challenge
**Feasibility studies**				
Arriola, Edvardsen, Solvoniemi, et al.^56^ 2023 Spain	Assess patients’ and HCPs’ use of digital patient monitoring tool in clinical practice	Theoretical framework = not mentioned Setting = 10 cancer centers in Estonia, Finland, Greece, Norway, Portugal, Spain, and Sweden Population = advanced or metastatic lung cancer (non–small cell lung cancer or small cell lung cancer) or HER2-positive breast cancer Sample size = 153 patients and 70 HCPs Age = 62% of patients were 60 y and older Female = 45% Race and ethnicity = not mentioned Treatment type = anti–PD-L1 agents, other immunotherapy, combination therapies	PROMs = 26-item CTCAE Mode = Kaiku health web-based application Duration = 15 months Frequency = weekly Clinician summary report = yes, daily Patient summary report = yes Alerts to clinicians = yes Alerts to patients = yes Patient education = yes	Adoption (percentage of patients who accepted the invitation from their clinicians) = 85% Weekly adherence = 76%-81% User (patient and clinicians) experience positive; however, the time spent using the tool each week was low
Msaouel, Oromendia, Siefker-Radtke, et al.^46^ 2021 USA	Provide the accurate information to inform specific remedies for immune toxic effects in patients treated with ICIs	Theoretical framework = not mentioned Setting = MD Anderson Cancer Center Population = genitourinary cancer Sample size = 50 Median age = 65 y Female = 17% Race and ethnicity = not mentioned Treatment type = PD-1 inhibitor 91%, PD-L1 inhibitor 2.2%, CTLA-4 inhibitor 24%, interleukin-2 pathway therapy 31%, tyrosine kinase inhibitor 29%, cytotoxic chemotherapy 4.4%, combination 82%	PROMs = 16 items PRO-CTCAE inspired but developed by authors Mode = smartphone application Duration = median 63 days (range = 35.5-122 days) Frequency = at least 3 times per week Clinician summary report = yes Patient summary report = yes Alerts to clinicians = yes Alerts to patients = yes Patient education = not mentioned (advice provided by clinician when an alert was addressed)	Most frequently alerted symptoms= myalgia and arthralgia; 9% of alerts were linked to an adverse event and treatment course correction Alert thresholds were changed for 44% of symptoms (reducing stringency) When there was no alert triggered, there was no excess toxicity 95% of the time Of patients, 100% submitted questionnaires at least once a month Median time to respond to alerts was 19 hours; 89% of alerts were resolved within 7 days No increase in care staffing
Iivanainen, Alanko, Vihinen, et al.^41^ 2020 Finland	Investigate the feasibility of using PROMs, including symptoms reported, number and etiology of alerts, correlations between different symptoms and treatment benefit, and patient adherence	Theoretical framework = not mentioned Setting = 3 cancer centers Population = mixed advanced cancers Sample size = 37 Median age = 62 y Female = 27% Race and ethnicity = not mentioned Treatment type = anti–PD-L1 agents	PROMs = 18-item CTCAE Mode = Kaiku health web-based application Duration = 24 weeks Frequency = weekly Clinician summary report = not mentioned Patient summary report = not mentioned Alerts to clinicians = yes Alerts to patients = no Patient education = not mentioned	Patient-reported symptoms and severity Most common grade 1-2 symptoms= fatigue, cough, pain in joints, itching, loss of appetite, nausea, shortness of breath Most common grade 3-4 symptoms = cough, loss of appetite, nausea Alerts 67 alerts, common reasons were loss of appetite and shortness of breath, pain in joints, blurred vision, and cough. Alerts were for unknown reasons (57%), side effects (31%), and cancer progression (11%) Patient adherence Patients completed close to 1 PROM per patient per week. Survey results= 90% ease of use, 90% improved care, 95% would recommend to others
**Observational**				
Iivanainen, Alanko, Peltola, et al.^40^ 2019 Finland	Compare symptoms collected via PRO tool with those reported in clinical trials	Theoretical framework = not mentioned Setting = Docrates Cancer Center, Oulu University Hospital Population = cancer mixed Sample size = 37 Median age = 61 y Female = 35.1% Race and ethnicity = not mentioned Treatment type = anti–PD1, anti–PD-L1, and anti–CTLA-4	PROMs = 19-item CTCAE plus EORTC QLQ-C30 Mode = Kaiku health Duration = 24 weeks Frequency = weekly for PRO-CTCAE and 1-2 months for EORTC-QLQ-30 Clinician summary report = not mentioned Patient summary report = not mentioned Alerts to clinicians = yes Alerts to patients = no Patient education = not mentioned	Adherence = PRO-CTCAE more likely to be completed than EORTC-QLQ-C30, adherence highest at weeks 3-4 Electronic PRO tool and clinical trial data = symptom variety, grading, and incidence matched clinical trial information Correlation of symptoms = strongest correlations between itching and rash, nausea and vomiting Low QOL correlated with fatigue, decreased appetite, nausea and dizziness
**Protocols**				
Fraterman, Wollersheim, Tibollo, et al.^61^ 2023 Netherlands	Evaluate the Cancer Patients Better Life Experience app in providing symptom monitoring, education, and well-being interventions on QOL, as well as its acceptability and usability	Theoretical framework = not mentioned Setting = Netherlands Cancer Institute Population = patients with stage III or IV melanoma Sample size = 139 Treatment type = anti–PD 1 or anti–CTLA-4	PROMs = 130-item list derived from PRO-CTCAE and CTCAE plus distress thermometer plus the Patient Health Questionnaire (PHQ-9) plus General Anxiety Disorder (GAD-7) plus wearable smartwatch Mode = Cancer Patients Better Life Experience app Duration = 3-6 months Frequency = not mentioned Clinician summary report = yes Patient summary report = not mentioned Alerts to clinicians = yes Alerts to patients = not mentioned Patient education = yes	Not applicable
Da Silva Lopes, Colomer-Lahiguera, Darnac^59^ 2023 Switzerland	Assess the effect of PROMs on irAEs onset, severity, and detection; health-related QOL; self-efficacy; and; overall survival at 6 months	Theoretical framework = not mentioned Setting = 2 Swiss university hospitals Population = patients treated with cancer receiving ICI Sample size = 198 Treatment type = ICI	PROMs = 37 items from PRO-CTCAE plus patient triggered additional PRO-CTCAE items Mode = Kaiku Health Duration = 6 months Frequency = weekly, daily for active symptoms first 3 months Clinician summary report = yes Patient summary report = yes Alerts to clinicians = yes Alerts to patients = not mentioned Patient education = yes	Not applicable
Iivanainen, Baird, Balas, et al.^57^ 2023 Finland	Impact on health outcomes and health-care usage of digital patient monitoring and its feasibility in supporting at-home treatment administration	Setting = 40 sites across 10 countries Population = non–small cell lung cancer (advanced or early stage) and liver cancer Sample size = 400 Treatment type = atezolizumab and combination therapy	PROMs = 24 PRO-CTCAE items tailored to atezolizumab Mode = Kaiku Duration = 18 months Frequency = weekly plus day after treatment Clinician summary report = yes Patient summary report = yes Alerts to clinicians = yes Alerts to patients = yes Patient education = yes	Not applicable
Kestler, Kühlwein, Kraus, et al.^45^ 2021 Germany	Investigate the use of a digital approach and CTCAE-adapted questions for patient-reported symptoms	Theoretical framework = not mentioned Setting = University Hospital Ulm Population = received treatment Sample size = 30 for feasibility study, 36 for reduction of adverse events Treatment type = not mentioned	PROMs = 21-item CTCAE based Mode = NEMO (German, Nebenwirkungs-management Onkologie) phone application Duration = 6 months Frequency = daily Clinician summary report = yes, every 2 months Patient summary report = yes Alerts to clinicians = No Alerts to patients = no Patient education = not mentioned	Not applicable
Sauer, Krauß, Jäger, et al.^47^ 2021 Germany	Assess the feasibility of SOFIA (managing **s**ymptoms **ofi**mmunother**a**py), which includes PRO monitoring and coaching for the management of symptoms	Theoretical framework = not mentioned Setting = National Center for Tumour Disease, Heidelberg Population = any cancer type starting ICI Sample size = 70 Treatment type = ICI monotherapy and combination therapy	PROMs = 20-item PROM based on European Society for Medical Oncology (ESMO) guidelines and Distress Thermometer (DT) (PHQ-4, if DT ≥ 5) Mode = SOFIA phone application Duration = 3 months Frequency = twice a week Clinician summary report = yes Patient summary report = no Alerts to clinicians = no Alerts to patients = yes Patient education = yes	Not applicable
**Machine-learning studies**				
Iivanainen, Ekstrom, Virtanen, et al.^42^ 2021 Finland	Determine the prognostic value of PROs in predicting the presence and onset of irAEs	Included 34 patients from Iivanainen, Alanko, Vihinen, et al.^41^	Same as Iivanainen, Alanko, Vihinen, et al.^41^	Machine learning models based on the electronic PROMs could accurately predict the presence of irAEs. The machine learning models had a good level of discrimination in predicting the onset and continuation of irAEs
Iivanainen, Ekstrom, Virtanen, et al.^44^ 2020 Finland	Prognostic value of PROs in predicting whether symptoms will onset or continue in the upcoming days or they will not onset or continue	Original dataset = 21 744 reported symptoms from 72 ICI patients. Data split into 2 = 70% for training and tuning and 30% for initial validation of the models Test dataset = 16 884 reported symptoms from 67 cancer patients	Not described in detail; 18 PROs included, similar to Iivanainen, Alanko, Vihinen, et al.^41^	Overall performance of the models was good. Most predictive PROs were dyspnea, joint pain, cough, and fatigue
Iivanainen, Ekström, Virtanen, et al.^43^ 2022 Finland	Examine the prognostic value of PROs in predicting treatment response	Included 34 patients from Iivanainen, Alanko, Vihinen, et al.^41^	Same as Iivanainen, Alanko, Vihinen, et al.^41^	The 2 most important symptoms for predicting treatment response were itching and fatigue, in combination with gender and lab results (eg, bilirubin)
**Description of a PRO program**				
Da Silva Lopes, Colomer-Lahiguera, Darnac^58^ 2023 Switzerland	Develop a model of care leveraging electronic PRO data to monitor and manage symptoms of patients treated with ICI	Theoretical framework = Enhanced Chronic Care Model Setting = outpatient care Population = treated with ICI Sample size = 5 nurses, 4 physicians, 2 patient representative Mean age = not reported Female = not reported Race and ethnicity = not reported Treatment type = ICI	PROMs = 37 items from PRO-CTCAE plus patient triggered additional PRO-CTCAE items Mode = Kaiku Health Duration = 6 months Frequency = weekly, daily for active symptoms Clinician summary report = yes Patient summary report = yes Alerts to clinicians = yes Alerts to patients = not mentioned Patient education = yes	Not applicable
Yanez, Bouchard, Cella, et al.^54^ 2019 USA	Description of OncoPRO and OncoTool	Theoretical framework = combination, including biopsychosocial model, theories of stress and coping, models of chronic care, and self-management Setting = not mentioned Population = patients with advanced cancer Sample size = not mentioned Treatment type = includes immunotherapy	PROMs = PRO-CTCAE and additional items Mode = using web platform OncoPRO and OncoTool Duration = not mentioned Frequency = not mentioned Clinician summary report = not mentioned Patient summary report = yes Alerts to clinicians = yes Alerts to patients = not mentioned Patient education = yes	Not applicable

a T = C, no significant difference between treatment and control; T > C, treatment on reported outcome greater or higher than control; T < C, treatment on outcome lower or less than control. C = control; CTCAE = Common Terminology Criteria for Adverse Events; CTLA-4 = cytotoxic T-lymphocyte associated protein 4; EORTC-QLQC30 = European Organisation for Research and Treatment on Cancer Quality of Life Questionnaire Core 30; HCP = health-care professional; ICI = immune checkpoint inhibitor; irAE = immune-related adverse event; PD-L1 =  programmed cell death protein 1 ligand; PRO = patient-reported outcome; PROM = patient-reported outcome measure; QOL = quality of life; RCT = randomized controlled trial; T = treatment.

### Participants

Two-thirds of studies had a sample of less than 100 patients.^40-48^^,^^52^^,^^53^ Slightly more than half of studies included patients with mixed cancer diagnoses,^40-44^^,^^46^^,^^53^^,^^55-57^^,^^59^^,^^60^ and remaining studies focused on patients with melanoma.^49-52^^,^^61^ All 3 types of immune checkpoint inhibitors were included: anti–PD-1^40^^,^^46^^,^^60^^,^^61^ and anti–PD-ligand 1 agents,^40^^,^^41^^,^^46^^,^^48^^,^^50-53^^,^^56^^,^^57^ and CTLA-4 inhibitors.^40^^,^^46^^,^^50-52^^,^^60^^,^^61^ Some studies included patients on combination therapies.^46^^,^^50-52^^,^^56^^,^^57^^,^^60^

### Theoretical frameworks

Theoretical frameworks were explicit in 2 studies, including a combination of theories (eg, biopsychosocial model, theories of stress and coping, and models of chronic care and self-management)^54^ or the Enhanced Chronic Care Model.^58^

### Real-world implementation

Seven studies labeled as “real-world”^40^^,^^44^^,^^48^^,^^53^^,^^56^^,^^58^^,^^60^ did not explicitly define the term but focused on PROM implementation within existing health-care resources. These studies included adapting a PROM platform already in place to the specific needs of patients receiving immune checkpoint inhibitors,^48^ implementing a new PROM program,^48^^,^^53^^,^^56^^,^^60^ retrospective analysis of data collected through a PROM platform in place,^40^^,^^44^ or development of a PROM data collection model for real-world use.^58^ None detailed specific implementation strategies. Few mentioned training clinicians in patient-centered communication^49^ or familiarizing them with the programs’ functionalities.^46^^,^^48^^,^^60^ One study described role-based training simulations encompassing patient onboarding, electronic PROM completion, result triaging, and clinician-patient communication.^48^

### Types of PROM programs

Ten PROM programs were developed specifically for patients receiving immunotherapy.^40^^,^^41^^,^^46-48^^,^^50-52^^,^^55^^,^^57-59^^,^^61^ Three programs were adaptable across various cancers and treatments and had developed PROMs and/or educational materials specific to immunotherapy.^45^^,^^54^^,^^56^ The remaining 3 programs were not specific to immunotherapy.^49^^,^^53^^,^^60^

### PROM program goals

The PROM programs’ main goal was remote monitoring of immune-related adverse events severity (or grade).^40-42^^,^^45-48^,^50-56^^,^^58^^,^^59^^,^^61^ Other goals included improving efficiency of symptom monitoring (eg, reducing follow-up time),^48^^,^^55^^,^^56^^,^^60^ standardizing symptom reporting,^53^ improving patient-clinician communication,^46^^,^^49-53^ and providing patient education.^47^^,^^54^^,^^56^^,^^57^^,^^61^

### Patient-reported outcomes screened for and PROMs used

Patients completed between 16^46^ and 130^61^ items, with half of the programs involving between 18 and 26 items. Three-quarters of programs used the PRO-CTCAE^46^^,^^49-51^^,^^57-60^ and/or CTCAE (adapted to be given to patients).^40^^,^^41^^,^^45^^,^^53^^,^^55^Table 2 summarizes the PROMs used, detailing corresponding symptoms. Some programs also included items and measures that were developed by the authors (eg, weight, fever).^45-47^ Patients could report any other symptom in an open field for 5 programs.^40^^,^^41^^,^^45^^,^^46^^,^^48^^,^^57^ Additional features that were available included uploading pictures;^55^ connecting smartwatch^61^ to collect data on heart rate, blood pressure, sleep, physical activity, and stress (data not used for symptom monitoring); sending messages and attachments to clinicians;^56^^,^^57^ or triggering additional PRO-CTCAE items.^59^

**Table 2.** List of symptoms screened for (when available)

SymptomsPrograms	Zhang et al.^55^	Iivainen et al.^a^^,40,41^	Kestler et al.	Msaouel et al.^46^	Sauer et al.^47^	Schmalz et al.^48^	Skovlund et al.^49^	Tolstrup et al.^50-52^	Trojan et al.^b^^,53^	Arriola et al.^56^	Iivainen et al.^57^
Alopecia								x			
Anxiety		x		x	x		x		x	x	x
Arrythmia	x										
Blood pressure			x								x
Blood in stool	x	x		x	x	x		x		x	
Blood in urine		x				x				x	x
Blurred vision	x	x		x		x		x		x	
Chills								x			
Concentration		x		x			x		x		
Constipation		x					x	x		x	x
Cough	x	x		x	x	x		x	x	x	x
Daily activities, difficulties		x					x				
Decreased appetite	x	x	x			x	x	x	x	x	x
Depression, mood change	x	x			x		x	x	x		x
Diarrhea	x	x	x	x	x	x	x	x		x	x
Distress					x						
Dizziness		x		x		x		x	x	x	
Dry mouth									x	x	
Dry skin								x			
Edema								x			
Eye discomfort	x										
Family life, difficulties		x									
Fatigue		x	x	x	x	x	x	x	x	x	x
Feeling swollen					x						
Feeling tense							x				
Fever		x		x	x	x			x	x	x
Financial difficulties		x					x				
Function		x					x		x		
Headache	x	x				x		x	x	x	x
Hemoptysis										x	x
Hot flashes								x	x		
Injection site reaction								x			x
Interference in daily activities		x		x			x				
Irritability		x					x				
Jaundice			x		x						
Memory		x		x	x		x				
Mucositis								x		x	
Nausea	x	x		x		x	x	x	x	x	x
Numbness, tingling								x		x	x
Pain, abdominal	x	x		x		x		x	x	x	x
Pain, general		x	x	x			x	x	x		
Pain, joint	x	x	x	x	x	x		x	x	x	x
Pain while eating			x								
Pain, chest	x	x				x			x	x	x
Pain, muscle			x	x	x			x			
Pain, urination				x							
Palpitations	x			x							x
Pulse			x								
Respiratory distress	x		x								
Sensibility			x								
Shortness of breath	x	x		x	x	x	x	x	x	x	x
Skin blisters, peeling	x										
Skin, itching	x	x		x	x	x		x		x	x
Skin rash	x	x	x	x	x	x		x	x	x	x
Skin redness	x		x		x						
Skin toxicities					x						
Sleep problems		x					x		x		
Stomatitis									x		
Swelling										x	x
Taste change								x	x	x	x
Thirst	x										
Urge symptoms									x		
Urination, reduced					x						
Urination, excessive	x										
Vision changes			x								
Vomiting		x	x	x		x	x	x		x	x
Walking, difficulties		x					x				
Weakness	x	x			x		x	x	x		
Weight			x							x	x
Well-being, quality of life		x	x				x		x		
Worry		x					x				

a Iivanainen et al.^41^ did not include the European Organisation for Research and Treatment of Cancer-Quality of Life Questionairre Core 30.

b A total 54 symptoms screened for but information for a subset available in the publications.^53^^,^^85^

#### Symptoms screened for by the PROM programs

A total of 71 symptoms were screened for across the PROM programs reviewed (see Table 2). Symptom selection was determined by clinical experts^45-47^^,^^56-59^ and/or based on literature reviews,^45^^,^^48^^,^^50-52^ interviews with patients,^48^^,^^57^ patients’ partners,^49^^,^^56^^,^^58^^,^^59^ interviews with clinicians,^48^^,^^57^ reviewing data from clinical trials,^40^^,^^41^ chart reviews,^50-52^ and/or product information.^40^^,^^41^^,^^50-52^^,^^56^^,^^57^ One program was based on a Delphi survey involving 11 clinical experts.^58^^,^^59^ Below, the most common symptoms screened for are summarized (presented in decreasing order) for the 10 programs that provided this information.

1. Gastrointestinal symptoms, including diarrhea,^40^^,^^41^^,^^45-53^^,^^55-57^ nausea,^40^^,^^41^^,^^46^^,^^48-53^^,^^55^^,^^56^ decreased appetite,^40^^,^^41^^,^^45^^,^^48-53^^,^^55^^,^^56^ vomiting,^40^^,^^41^^,^^45^^,^^46^^,^^48-53^^,^^56^^,^^57^ and abdominal pain^40^^,^^41^^,^^46^^,^^48^^,^^50-53^^,^^55^
2. Fatigue^40^^,^^41^^,^^45-53^^,^^56^^,^^57^ or sleep problems^40^^,^^49^^,^^53^
3. Shortness of breath and cough^40^^,^^41^^,^^46-48^^,^^50-53^^,^^55-57^
4. Pain,^40^^,^^45-47^^,^^49-53^ including in general and specific types of pain such as joint,^40^^,^^41^^,^^45-48^^,^^50-53^^,^^55-57^ chest,^40^^,^^41^^,^^48^^,^^53^^,^^55^^,^^57^ and/or muscle^45-47^^,^^50-52^
5. Anxiety and/or depression^40^^,^^41^^,^^46^^,^^47^^,^^49^^,^^53^^,^^56^^,^^57^
6. Rash^40^^,^^41^^,^^45-48^^,^^50-53^^,^^55-57^ and/or itching^40^^,^^41^^,^^46-48^^,^^50-52^^,^^55-57^
7. Weakness and lack of energy^40^^,^^47^^,^^49-53^
8. Fever^40^^,^^41^^,^^46-48^^,^^53^^,^^57^
9. Blood in stools^40^^,^^41^^,^^46-52^^,^^55^^,^^56^
10. Headaches^40^^,^^41^^,^^48^^,^^50-53^^,^^55-57^ and/or dizziness^40^^,^^41^^,^^46^^,^^48^^,^^50-53^

Of note, beyond anxiety or depression, the impact of physical symptoms on function, well-being, daily activities, and other difficulties (eg, financial) was measured in few programs.^40^^,^^41^^,^^45^^,^^46^^,^^49^ One program asked about exercise.^45^

Three studies examined the prognostic value of PROM data in predicting onset and presence of immune-related adverse events.^42-44^ Dyspnea, joint pain, cough, and fatigue were found to be most predictive of whether symptoms will onset or continue in the upcoming days.^43^ Diarrhea and joint pain were most predictive of the presence of immune-related adverse events,^42^ whereas fever and chest and stomach pain were more predictive of the future onset of immune-related adverse events.^42^ PROM data were also found to predict treatment response, with itching and fatigue as the 2 most important symptoms, in combination with gender and lab results (eg, bilirubin).^43^

### Administration of the PROMs

#### Frequency and duration

Slightly more than half of the programs required weekly monitoring.^40^^,^^41^^,^^48^^,^^50-52^^,^55-59^^ Other frequencies included 2 times a week,^47^ 3 times a week,^46^ or up to once a day.^45^^,^^53^ Acknowledging the burden of daily symptom reporting, Kestler et al.^45^ limited monitoring to symptoms with high impact on treatment safety. Da Silva Lopes et al.^58^^,^^59^ re-assessed only active symptoms daily for the first 3 months. Except 1 program,^49^ PROMs were triggered in between clinic visit to monitor toxicities closely. Reminders to complete the PROMs were common,^40^^,^^41^^,^^46^^,^^53-56^^,^^58^ though 1 program required patients to choose a fixed weekday to respond instead of reminders.^50-52^ Nine programs lasted 6 months,^40^^,^^41^^,^^45^^,^^51^^,^^55^^,^^58-61^ and 6 with weekly PROMs.^40^^,^^41^^,^^51^^,^^55^^,^^58^^,^^59^

#### Adherence

Weekly PROM adherence was high, ranging from 76% to 81%,^48^^,^^50^^,^^56^ with patients spending at most 10 minutes on the tool.^56^ Iivanainen et al.^40^ reported peak adherence in weeks 3-4 of their 24-week study using PRO-CTCAE weekly and the European Organisation for Research and Treatment of Cancer Quality of Life Questionnaire Core-30 every 1-2 months.^40^ However, adherence was lower for the European Organisation for Research and Treatment of Cancer Quality of Life Questionnaire Core-30 compared with PRO-CTCAE, with a median of 11 PROMs completed per patients (of approximately 34). In another study requiring PROMs at least 3 times a week, adherence remained high at 74%, with all patients completing at least 1 PROM per month.^46^ A quasi-experimental trial^60^ found 31% of patients used the patient-reported outcome platform, completing on average 56.9% of their PROMs. Users tended to be women, White, married, living with a spouse, and having a college or graduate degree. A study included data regarding use of the PROM program among clinicians (n = 27), revealing varied usage frequencies: 18.5% daily, 40.7% multiple times a week, 30% weekly, and 15% monthly.^48^

#### Format

All programs used an electronic format (web-based platforms or smartphone applications); 1 program offered a paper or electronic option.^50-52^ Web-based platforms were Ambuflex,^49-52^ Kaiku Health,^40^^,^^41^^,^^48^^,^^56-59^ and unnamed or custom built,^54^^,^^55^ whereas the smartphone applications included Cancer Patients Better Life Experience,^61^ Noona,^60^ NEMO,^45^ SOFIA,^47^ Consilium Care,^53^ and unnamed.^46^ In 1 study, the application was available on smartphone, tablet, or desktop, and all end users (patients, nurses, and physicians) preferred the desktop.^48^

### Integration into clinical workflow

#### Clinician summary report

PROM data were frequently presented to clinicians in longitudinal graphs,^40^^,^^41^^,^^45^^,^^47-54^^,^^58^^,^^59^ with 4 programs^49-51^^,^^53^^,^^58^^,^^59^ color-coding graphs based on symptom severity (eg, no symptom = green; mild symptom = yellow; moderate symptom = orange; severe symptom = red). One program featured a chat function enabling direct patient-clinician communication.^48^ For some programs, a physician or nurse was assigned to monitor the PROM data reports.^56^^,^^58^^,^^59^^,^^61^

Three programs integrated the clinician symptom report to the electronic medical record,^47^^,^^49^^,^^54^ though real-time integration was automatic only in 1 program.^49^ In others, reports were transcribed by a member of the research team (which occurred on the day of the appointment)^47^ or linked to the patient’s electronic medical record manually by the research staff.^54^ In other programs, clinicians had to log in a different web platform,^46^^,^^50-52^^,^^61^ received the reports by email,^46^ or accessed the report via a QR code on their desktop app.^45^

#### Patient summary report

In 7 programs, an overview of the PROM data was available to patients.^40^^,^^41^^,^^45^^,^^48^^,^^53^^,^^54^^,^^56^^,^^58^^,^^59^ Typically, patients accessed their symptom reports in the platform or app.^53^^,^^54^^,^^58^^,^^59^ None of the studies noted whether the patient reports differed from those for the clinicians or if any support was available to help the patients understand the information.

#### Alerts

In 5 programs,^48^^,^^54^^,^^55^^,^^57-61^ alerts were directed solely to clinicians, prompting them to contact patients to manage symptoms using available resources. In a feasibility study, clinicians identified alerts as the most useful feature of PROM programs, enabling early symptom detection.^48^ In 2 programs, only the patient received the alerts^47^^,^^50-53^ and were advised to contact their clinician. Two other programs alerted both clinicians and patients.^46^^,^^56^

Clinicians received alerts by email,^40^^,^^41^^,^^46^^,^^55^^,^^56^^,^^58^^,^^59^ electronic medical record in-basket system,^54^ text messages on their phone,^55^ or in-app notifications.^55^ Patients often received the alerts through in-app notifications.^46^^,^^47^^,^^53^ Alerts were commonly triggered for symptoms categorized as grades 3 or higher and/or if a symptom worsened by at least 2 grades.^40^^,^^41^^,^^46-48^^,^^55^^,^^56^ In 2 programs, alerts were only triggered for symptoms that could potentially become severe^50-52^ or could lead to a severe symptom soon (eg, fever).^46^ Symptom such as alopecia or fatigue did not trigger alerts.^50-52^ One program^46^ used specific severity thresholds for each symptom. For example, the threshold for nausea was “severe,” whereas the threshold for itchy skin was “very severe.” da Silva et al.^58^^,^^59^ described 3 types of alert: (1) green for mild symptoms where self-management support was recommended, (2) amber for symptoms needing reassessment because of changes, and (3) red for 2 or more amber alerts or a severe symptom requiring in-person assessment.

Two studies^41^^,^^52^ flagged discrepancies between the alerts received and then clinicians’ assessment. For instance, clinicians felt that patients neglected important symptoms or clinicians did not find the symptoms to be severe.^52^ In one of these studies, 67 alerts were triggered over 12 weeks, commonly for loss of appetite, shortness of breath, pain in joints, blurred vision and cough. Clinicians attributed 31% of the alerts to treatment side effects and 57% to “unclear reasons.”^41^ Another study reported a median response time of 19 hours for care teams to address alerts, with 73% reviewed within 3 days and 89% within 7 days.^46^ Symptoms like nausea, dizziness, and shortness of breath were most often addressed within 2 weeks of the alert.^46^ Although joint and muscle pain were not frequent, they accounted for the most alerts (9.45%), followed by pain (7.72%) and fatigue (5.33%). Only 9% of alerts were deemed appropriate (ie, those that led to interventions to mitigate toxicity), with dizziness, nausea and vomiting, and shortness of breath most likely leading to appropriate alerts.^46^ The symptoms more likely to result in unnecessary (as judged by the health-care team) alerts were joint and muscle pain, fatigue, and cough. Altogether, an intervention from the clinicians was required for 6.9% of the alerts (eg, withholding treatment, change in dose).^46^

#### Patient education

In 9 programs, patients received information to support symptom management, delivered directly through the platform or application.^47^^,^^48^^,^^53-58^^,^^61^ However, few details were provided about the patient education resources. One program offered a coaching app, which contained 24 self-management modules (eg, relaxation, coping, fatigue, information about social and financial support, nutrition, and physical activity).^47^ Schmalz et al.^48^ found that 80% (36 of 45) of patients engaged with the educational materials provided, which mostly included treatment-specific information along with a breathing video. Another program offered the OncoTool app including education about treatment toxicities and information on symptom and stress management, as well as a decision-making questionnaire.^54^ Fraterman et al.^61^ asked patients, in collaboration with clinicians, to set specific goals for symptom management and select interventions to achieve those goals.

### Outcomes of using PROM programs (excludes protocols)

#### Patient satisfaction

Patient satisfaction was generally high,^48^^,^^52^^,^^56^ and completing the PROMs was felt to be easy or very easy^41^^,^^56^ and not too time consuming.^52^ Weekly administration was found to be “just right.”^52^ In multicomponent PROM programs, patients identified the PROMs as most useful (95%), because it helped them monitor their symptoms, improved quality of care, and increased their feeling of being well taken care of.^48^ Although many patients felt PROMs were reviewed by clinicians, there was frustration when clinicians did not specifically discuss these during consultations, given the time patients had invested completing the PROMs.^52^ Of note, in 1 study, 17% of patients needed assistance using the platform.^41^ In another study,^50^ 75% of patients declined participation because of lack of computer skills or they felt completing PROMs was too demanding.

#### Clinician satisfaction

Clinicians’ satisfaction was also generally high,^48^^,^^52^^,^^56^ albeit improvements were always noted (eg, integration of tools with electronic medical record). Satisfaction was found to be lower among nurses than physicians.^56^

#### Patient-clinician communication

In surveys and interviews, patients and clinicians generally reported that PROMs facilitated and increased the efficiency of patient-clinician communication.^48^^,^^52^^,^^56^ PROMs heightened patients’ and clinicians’ awareness of symptoms^41^^,^^52^ and helped patients remember symptoms during consultations.^52^ PROMs helped prioritize the most acute problems,^48^^,^^52^ which can lead to tailored discussions with patients.^48^ Clinicians felt that patients were better prepared for appointments, and PROMs led some patients to call the clinic before symptoms got too severe.^52^ However, a quasi-experimental trial^49^ found no effect of PROMs on the efficacy of the patient-physician interaction.

#### Patient activation

This same quasi-experimental trial^49^ found that although participants in the PROM program reported higher patient activation than those in the control group, differences were not statistically significant. Although in another study, patients reported that PROMs helped them feel more in control and helped them feel more secure during treatment and in evaluating their symptoms.^48^ In this same study, patient education was valued by clinicians and patients but less so than other features such as alerts for clinicians and the PROMs for patients.^48^

#### Clinician workload

In a feasibility study, clinicians spent on average 1-3 minutes per patient on the tool weekly.^56^ Mostly attributed to the aforementioned benefits on patient-clinician communication, a study reported that 44% of clinicians felt PROMs saved them up to 10 minutes.^48^ Similarly, a RCT found that the mean time spent in follow-up visits was 8.2 minutes (3.9 minutes, 95% confidence interval [CI] = 5.0 to 10.6 minutes) when PROMs were used vs 36.1 minutes (15.3 minutes, 95% CI = 33.6 to 38.8 minutes; *P* < .001) in usual care.^55^ In a feasibility study, alert resolution (ie, clinicians contacted patients when alerts were triggered) was not a major time burden, and additional staffing was not required because standardization of symptom screening facilitated automatic triaging.^46^ However, in another study, 26% of clinicians felt PROMs did not save time.^48^ The main negative impact of PROMs on workload was due to the extra time required to manage the platforms and entering data, especially in cases where there was no integration with the electronic medical record.^52^

##### Quality of life

Two RCTs^51^^,^^55^ and 1 quasi-experimental trial^49^ found PROMs had positive significant effects on QOL. One of these found no difference in QOL at 24 weeks, but at 48 weeks, QOL was statistically significantly higher for those in the PROM program group than the control group.^51^ Another RCT also found no differences in QOL at the first data collection time point (3 months), but there was a difference over time (6 months).^55^

#### Reduction of immune-related adverse events

Although a RCT showed a reduced incidence of serious immune-related adverse events in the PROMs program group compared with the control group,^55^ another^50^ found no difference. Of note, their PROM algorithms differed, with only 11 symptoms overlapping (see Table 2). Another difference is that in the effective RCT,^55^ patients could also upload pictures of examination results.

#### Health-care service utilization

In the RCT by Zhang et al.,^55^ emergency room visits were lower among those followed with PROMs in comparison with those who were not. In another RCT,^50^ the number of phone calls to the hospital were higher for the PROM group vs usual care; there were no differences in hospitalization, days in hospital, or extra visits to the hospital (including emergency room visits).

##### Survival

Two studies reported on survival. One RCT^55^ found no difference in death rates among those followed with PROMs in comparison with those who were not, whereas a quasi-experimental study^60^ found that the estimated 6-month overall survival was 72.4% among the PROM group vs 65.5% in the historical control group (*P* < .01).

#### Barriers and facilitators of patient-reporteds use (as reported by authors)

Key facilitators included clinicians and patients recognizing PROMs’ added value,^41^^,^^48^^,^^51^^,^^55^^,^^56^ integration in current workflows,^46^^,^^48^^,^^49^ seamless user experience (eg, optimization of user interface),^24^^,^^41^^,^^48^ organizational commitment,^60^ clinical informatics involvement,^60^ consistent branding for credibility,^60^ comprehensible items,^60^ PROM data mimicking physician-assessed symptoms and correlating with treatment benefits,^41^ and immune checkpoint inhibitor–specific items.^43^^,^^44^^,^^50^^,^^60^

A clinician barrier included interoperability issues (eg, no integration of PROM data in electronic medical records).^48^^,^^55^^,^^56^ For patients, barriers included high burden (eg, high frequency),^24^^,^^56^ needing assistance in using the platform,^41^ lack of computer literacy and online access,^60^ lower education,^60^ wariness about sharing personal information,^60^ too ill to answer,^51^ and lack of clinical follow-up on the results.^51^

## Discussion

The interest of using PROMs among those receiving immune checkpoint inhibitors is growing fast, and the goal of this scoping review was to provide an overview of the evidence to inform the development and evaluation of PROM programs.

Although the evidence base for the added value of PROMs among patients receiving immune checkpoint inhibitors is developing, a few RCTs and/or quasi-experimental studies corroborated some of the known benefits^19^^,^^62-67^^,^^68^ of using PROMs, including reduced workload^55^ and improving QOL.^49^^,^^51^^,^^55^ Findings for most of the other outcomes were mixed, including whether PROMs can reduce immune-related adverse events and health-care utilization and improve survival.^50^^,^^55^ RCTs are therefore needed to further develop this evidence base. Potentially innovative experimental designs could be considered (eg, Sequential Multiple Assignment Randomized Trial^69^) to answer questions about which components of a complex PROM program are needed.

The main goal of PROM programs reviewed was to monitor and identify severe toxicities in between appointments. The focus was therefore on physical symptoms, and beyond anxiety and/or depression, there was little to no focus on patients’ psychosocial concerns or daily functioning. For instance, despite the high cost of immune checkpoint inhibitors, only 2 programs screened for financial difficulties.^40^^,^^41^^,^^50-52^ This shifts how PROMs have been typically used away from understanding patient’s subjective experience of treatments toward remote monitoring of treatment toxicities. To this end, several programs^41^^,^^45^^,^^55^ developed measures from the clinician’s version of the CTCAE rather than using the patient facing PRO-CTCAE.^70^ Recently, grading scales using PRO-CTCAE have been validated and can be seen as complementary to clinician grading.^71^ Potentially, this shift reflects the newness of immune checkpoint inhibitors and the concern for patient safety, and future programs need to re-integrate psychosocial concerns.

The top 10 patient-reported outcomes included across programs were diarrhea, fatigue, shortness of breath, joint pain, cough, decreased appetite, nausea, rash, depression, and pain (general). Six of these overlap with the findings of Iivanainen et al.,^42^^,^^43^ who identified dyspnea, cough, itching, fatigue, diarrhea, and joint and stomach pain as most predictive of the onset or presence of immune-related adverse events and treatment response, in addition to fever and chest pain. When comparing this list with patient-reported outcomes captured in the 2 RCTs examining the impact on grade 3-4 immune-related adverse events,^50^^,^^55^ the RCT showing a statistically significant effect only lacked fever,^55^ whereas the RCT with no significant difference missed fever, fatigue, and chest pain.^50^ This difference and that patients could upload pictures of examination results^55^ point to key PROM program features for future studies.

Iivanainen et al.^41^ found that PROMs corroborated symptom profiles similar to those found in immune checkpoint inhibitor clinical trials but with higher prevalence. In clinical trials, clinician-based grading of immune-related adverse events might underestimate occurrence of immune-related adverse events compared with PROMs, especially low ones.^41^ In an immune checkpoint inhibitor clinical trial, the agreement between PRO-CTCAE and clinical grading using CTCAE ranged from a Cohen kappa of 0.10 (no agreement) to 0.64 (good agreement).^72^ Pruritus had the lowest agreement, whereas rash had the highest agreement, potentially because it is an observable symptom.

The most common PROMs in the studies reviewed were the CTCAE and PRO-CTCAE. Internationally, in clinical practice, the Edmonton Symptoms Assessment Scale-revised (ESAS-r) is the PROM often used.^73^ The ESAS-r and PRO-CTCAE have different response sets and recall periods, making their combination difficult. This potentially poses a challenge for real-world implementation, where centers have invested in using the ESAS-r (eg, automated fields in electronic medical records). To build on, instead of replacing what has already been implemented, a 2-stage screening algorithm could be considered, whereby a core set of patient-reported outcomes is explored with 1 PROM (ie, ESAS-r) and immune checkpoint inhibitor–specific symptoms are further screened using other tailored PROMs. This could be the PRO-CTCAE or others (eg, immunotherapy module of the MD Anderson Symptom Inventory,^74^ Functional Assessment of Cancer Therapy-Immune Checkpoint Modulator^75^).

Consistent with the PROM European Society for Medical Oncology Clinical Practice Guidelines,^76^ several programs included clinician alerts for severe or worsening symptoms. A study found that only 9% of alerts led to interventions to mitigate toxicity, with dizziness, nausea and vomiting, and shortness of breath most likely leading to appropriate alerts.^46^ Interestingly, Billingy et al.^77^ (not included in the review, 30% of patients received immunotherapy) tested 2 types of alerts among patients with lung cancer: active (alerts sent to clinicians) vs reactive (only patients receive the alert). Both types of alerts were equally effective on QOL, resulting in statistically significantly higher QOL in both groups than those in usual care. Potentially, the reactive approach can facilitate sustainable PROM implementation.^77^ Another consideration is to trigger alerts only for symptoms that could impact treatment or might become life-threatening. This is like the RCT by Tolstrup et al.,^50^^,^^51^ which found a significant improvement in QOL but not on incidence of immune-related adverse events, potentially because alerts were only sent to patients. This indicates that some clinician alerts are required.

Other than alerts, the PROM European Society for Medical Oncology Clinical Practice Guidelines^76^ suggest clinician training on how to review and interpret PROMs data. Such training was mentioned by few studies,^46^^,^^48^^,^^49^ and it was even less clear how teams responded to the PROM data (eg, what protocols were used). This might explain the mixed efficacy of PROM programs identified in this review.

Certain features of PROM programs might also affect patient engagement and then the extent to which PROMs were efficacious. Although some programs offered patients a summary of their PROMs data, this was less frequent than for clinicians. Patient information about symptoms management was included in 11 of 16 programs. A systematic review of electronic symptom reporting systems developed for patients during cancer treatment found that fewer than half included a feature for delivering advice to patients for symptom management.^78^ Only 1 program included a feature for patients to communicate with the health-care team, and none described other communication features (eg, forum for patients to exchange).

Seven studies^40^^,^^44^^,^^48^^,^^53^^,^^56^^,^^58^^,^^60^ focused on real-world implementation of PROM programs; however, none described the use of implementation science frameworks or implementation strategies and/or processes. Implementation strategies are techniques/actions to address implementation barriers and enhance the adoption of innovations.^79^ The barriers/facilitators to PROM programs identified across studies reviewed are not unique to the immune checkpoint inhibitor context.^80-82^ There is a conspicuous gap though in the use of implementation strategies to address the pervasive barriers to PROM implementation. This underscores an untapped opportunity to increase the success of PROM implementation.^83^ Newer hybrid trials^84^ would allow for the evaluation of implementation outcomes and strategies alongside effectiveness.

A limitation is that papers published in languages other than English or French were excluded. Some publications provided limited information about their PROM programs, but all authors were contacted, and if additional was information was received, it was integrated. Additional papers may be available by the time this review is published; however, we did include protocols to ensure the latest research was included, and the database search was updated in January 2024.

Patients receiving immune checkpoint inhibitors may benefit from PROMs to the same extent as those undergoing other therapies. This scoping review can guide the evidence-based development of PROM programs for patients receiving immune checkpoint inhibitors. Our findings caution on an overreliance on PROMs to determine the grade of immune-related adverse events. Rather, it is important to keep with the original intent of PROMs in reporting the subjective experience of patients. Whether the PRO-CTCAE or another PROM is used, the more critical question is how these will be integrated in usual care and clinicians’ workflow.

## Data Availability

Data extraction available upon request.
